# Laboratory Findings in Patients with Probable Dengue Diagnosis from an Endemic Area in Colombia in 2018

**DOI:** 10.3390/v13071401

**Published:** 2021-07-19

**Authors:** Jenny C. Cardenas, Sandra Y. Giraldo-Parra, Maria U. Gonzalez, Lady Y. Gutierrez-Silva, Lucy Jaimes-Villamizar, Alba L. Roa-Parra, Daisy J. Carvajal, Hugo O. Valdivia, Juan F. Sanchez, Tonya M. Colpitts, Berlin Londono-Renteria

**Affiliations:** 1 Vector Biology Laboratory, Department of Entomology, Kansas State University, Manhattan, KS 66505, USA; carocardenasg@hotmail.com (J.C.C.); maubago94@gmail.com (M.U.G.); yuvey716lagusi1982@hotmail.com (L.Y.G.-S.); lucyjv2009@hotmail.com (L.J.-V.); albalurp19@gmail.com (A.L.R.-P.); daisyjc41@hotmail.com (D.J.C.); 2 Clinical Laboratory, Los Patios Local Hospital, Los Patios, Norte de Santander 547079, Colombia; sandrayomara@hotmail.com; 3 Clinical Laboratory, Hospital Universitario Erasmo Meoz, Cucuta, Norte de Santander 547079, Colombia; 4 Clinical Laboratory, Hospital Emiro Quintero Cañizares, Ocana, Norte de Santander 547079, Colombia; 5 Clinical Laboratory, Hospital Jorge Cristo Sahium, Villa del Rosario, Norte de Santander 547079, Colombia; 6 Department of Microbiology, Universidad de Pamplona, Pamplona, Norte de Santander 547079, Colombia; 7 Department of Parasitology, U.S. Naval Medical Research Unit No. 6 (NAMRU-6), Callao 07001, Peru; hugo.o.valdivia.ln@mail.mil (H.O.V.); juan.sanchez209.ln@mail.mil (J.F.S.); 8 National Emerging Infectious Diseases Laboratories, Department of Microbiology, Boston University School of Medicine, Boston, MA 02128, USA; tmcol@bu.edu

**Keywords:** dengue virus, rapid diagnosis, hemogram, PCR

## Abstract

As demonstrated with the novel coronavirus pandemic, rapid and accurate diagnosis is key to determine the clinical characteristic of a disease and to improve vaccine development. Once the infected person is identified, hematological findings may be used to predict disease outcome and offer the correct treatment. Rapid and accurate diagnosis and clinical parameters are pivotal to track infections during clinical trials and set protection status. This is also applicable for re-emerging diseases like dengue fever, which causes outbreaks in Asia and Latin America every 4 to 5 years. Some areas in the US are also endemic for the transmission of dengue virus (DENV), the causal agent of dengue fever. However, significant number of DENV infections in rural areas are diagnosed solely by clinical and hematological findings because of the lack of availability of ELISA or PCR-based tests or the infrastructure to implement them in the near future. Rapid diagnostic tests (RDT) are a less sensitive, yet they represent a timely way of detecting DENV infections. The purpose of this study was to determine whether there is an association between hematological findings and the probability for an NS1-based DENV RDT to detect the DENV NS1 antigen. We also aimed to describe the hematological parameters that are associated with the diagnosis through each test.

## 1. Introduction

Dengue is a vector-borne viral disease that is a major public health problem in tropical and subtropical regions of the globe. According to the World Health Organization, the incidence of dengue has grown globally, reaching up to 4.2 million cases in 2019 [1]. Out of all these cases, 73% were reported in the Americas, and 25,000 of them were classified as severe cases and represented the main cause of mortality in children under 15 years of age in Latin American [1,2]. 

Dengue fever is caused by four distinct dengue virus serotypes (DENV1, 2, 3, and 4) that are comprised within the Flaviviridae family [3]. Each of these serotypes causes clinical disease that can range from mild undifferentiated fever to more severe and potentially life-threatening symptoms such as dengue hemorrhagic fever (DHF) and dengue shock syndrome (DSS) [3,4,5]. Interestingly, almost 70% of DENV infections may present themselves as asymptomatic [6]. Clinical characteristics of a mild infection with DENV include fever, rash, headache, and chills, and it is often indistinguishable from other infectious diseases that are also endemic in the tropics, including malaria or Zika fever [7,8]. Severe forms of DENV infection are characterized by an increase in vascular permeability, hypovolemia, and petechiae, which may lead to death within 24 h if not treated effectively. However, early diagnosis seems to have a significant positive impact on patient recovery when following appropriate treatment [9].

DENV is transmitted via the infectious bite of female *Aedes* mosquitoes, and mosquito surveillance as well as vector control remain as the main tools to combat this disease [10]. In Colombia, DENV is transmitted by two species of *Aedes* mosquitoes with *Ae. aegypti* as the primary vector in almost 80% of the Colombian territory, and *Ae. albopictus* as secondary vector in places where these species have been recently introduced [11,12]. Colombia is classified as hyperendemic for dengue transmission since all four serotypes are circulating throughout the country and are even within the same endemic foci [13]. Severe dengue presentations are usually associated with secondary infections caused by a different DENV serotype than the one causing the primary infection. Therefore, people living in hyperendemic areas, such as Colombia, are at higher risk of severe disease. 

In 2019, Colombia presented 127,553 dengue cases, which represents an increase of 185% from the 44,825 cases reported in 2018 [14]. In rural areas where access to health care is precarious, health personnel rely on clinical characteristics to diagnose febrile diseases due to increased costs, limited access to diagnostic methods, or increased time to diagnosis [15]. In the case of dengue fever, lower platelets with leukocytosis are often used to discriminate between dengue and other febrile diseases. Data from the Pan-American Health Organization (PAHO) shows that only 44.6% (1,415,658) of all dengue cases reported in 2019 in the Americas were laboratory confirmed [16]. In addition, natural disasters and social instability provoking forced migration, both within and between countries, have an impact on transmission, moving pathogens from one side of the border to the other and weakening the already fragile health infrastructure in developing countries [17]. Such an example is the current mass migration from Venezuela impacting almost every country in Latin America. In this case, access to health care and the availability of proper tools to identify infected people have become important issues [18]. 

In this scenario, rapid, accurate and cost-efficient diagnosis of dengue fever becomes critical for clinical care since it can prevent evolution to severe clinical presentations. Furthermore, accurate and prompt detection of DENV infections can guide vector control activities and can provide important data to guide public health policy, vaccine research, and monitor the effectiveness of control measures [15,19]. This situation demands the need to develop and test field deployable diagnostic tools. The development of rapid diagnostic tests (RDT) in the dipstick format has allowed the timely detection of DENV infections in areas with no access to electricity [20] although the sensitivity or specificity of these tests may be impacted by the presence of other flaviviruses in the area, among other factors [21,22]. Currently, DENV non-structural protein 1 (NS1) based tests have been developed into the dipstick format to offer the possibility of rapid and reliable diagnosis of dengue fever [23]. The DENV NS1 protein is abundant in serum during the acute phase of infection with DENV, and it is mainly detected during the first five to six days after the onset of fever [24,25].

In the absence of a specific treatment to cure dengue fever and prevent complications, an effective vaccine is urgently needed. Dengvaxia^®^ is the only vaccine commercially available to prevent DENV infection [26]. However, due to potential adverse effects in naïve populations, the vaccine is recommended for people older than nine years of age who have had previous exposure to DENV [26,27]. There is an important knowledge gap addressing the clinical characteristics of dengue fever that may be associated with the performance of diagnostics test. The characterization of infection status and hematological findings during DENV infection in endemic areas will not only help in the design of improved vaccines but will also help in the identification of at-risk age groups as well as provide tools for early identification of infected individuals after vaccine deployment in the field [28]. Thus, with this study, we wanted to answer the question: what hematological parameters are characteristic of people who are diagnosed with dengue fever by clinical symptoms and how do those parameters correlate with diagnostic tests in hyperendemic areas? To answer this, we compared hemogram results among people diagnosed as a “probable dengue” case using clinical parameters and performed an NS1-based rapid diagnostic test (RDT) and reverse transcription polymerase chain reaction (RT-PCR) to detect current DENV infections. 

## 2. Materials and Methods

### 2.1. Study Area

**Figure 1:**
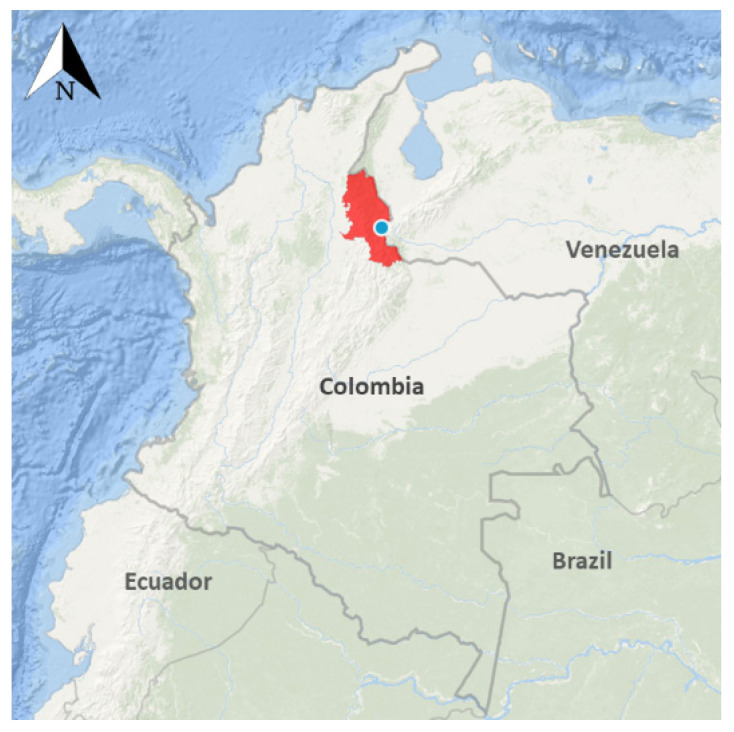
Map of the study site located the State of Norte de Santander, Colombia.

The protocols and methods for this study were reviewed and approved by the Kansas State University Ethics Review Board (IRB#8753) and The Los Patios Hospital Board. This study was conducted in Los Patios, in the State of Norte de Santander, which is located in the northeast of Colombia (Figure 1). Volunteers seeking medical attention in the hospital due to febrile illness and referred to the clinical laboratory for further hematological testing were approached by a research team member and asked to participate in the study. The objectives of the research were clearly explained to each potential participant (legal guardian or parent for children) and written informed consent was obtained prior to sample collection. A blood sample was only collected from people who agreed to be part of the study. In our current study, a “probable dengue case” is defined as a person with clinical symptoms and hematological findings compatible with dengue fever but without a confirmatory test. Furthermore, a person is classified as a “confirmed dengue case” when a diagnostic test (RDT or molecular test) is found to be positive for DENV infection.

### 2.2. Human Sample Collection, Blood Testing and Dengue Diagnosis

Blood samples (5 mL each) were collected from all of the voluntary participants reporting between 3 to 15 days of DENV-like symptoms and seeking medical care at the Los Patios (Los Patios Hospital) between January to December of 2018. Whole blood was used to determine leucocyte count, erythrocyte count, hemoglobin, hematocrit (HTC), platelet count, and the percentage of lymphocytes, monocytes, neutrophiles, eosinophils, and basophils using the hematology analyzer Celltac MEK-6800. Each blood sample was then centrifuged, and plasma was collected for use in a DENV (NS1)-based RDT or IgM ELISA. Specifically, the Xerion DENGUE antigen (Ag) (DENV-NS1-Ag) (Xerion—IMEX group, Bogota) was used to determine DENV infection following factory recommendations. In brief, shortly after the collection of the human plasma, the DENV-NS1-Ag Cassette and dropper were carefully removed from the aluminum packaging and identified with the patient code. With the dropper upright, the plasma sample was collected and transferred to the absorbent orifice of the cassette (approximately 30 μL plasma). One (1) drop of buffer (~30 μL) was then added to the absorbent orifice of the cassette, preventing bubbles from forming. Results were recorded after 20 min of incubation following the manufacturer’s recommendations. The remaining plasma was stored at −20 °C until it was shipped to the vector biology laboratory at Kansas State University (Manhattan, KS, USA). RNA was later extracted and used to detect the presence of the viral genome by quantitative, reverse-transcriptase polymerase chain reaction (qRT-PCR), which was performed using the conditions and primers published by our group elsewhere [25,27,29].

### 2.3. Statistical Analysis

Statistical significance, sensitivity, and specificity of the DENV-NS1-Ag test in comparison to the PCR-based test was determine using Fisher’s exact test. The difference between two independent groups (i.e., leukocyte count between DENV-NS1-Ag positive and negative test subjects) was determined using the Mann–Whitney test, with a *p*-value < 0.05. Comparison of more than three independent groups was tested with the Kruskal–Wallis test. Correlation analysis between to independent parameters was done using the Spearman correlation method. Statistical analysis was performed using GraphPad Prism, version 7 (GraphPad Software Inc., La Jolla, CA, USA). 

## 3. Results

### 3.1. Children under 15 Represent the Higher Number of Dengue Positive Cases by RDT

From January to December 2018, 161 participants were included in this study. Fifty-seven percent (92/161) of patients with probable dengue fever diagnosis who attended the Hospital Local de Los Patios were female, and 78% (126/161) of them were 15 years old or younger (Table 1). From the study sample, 64% (103/161) presented a positive DENGUE NS1-Ag.

In addition, we observed a significant difference in the median age between DENV-NS1-Ag test positive and negative volunteers with probable dengue fever diagnosis. DENV-NS1-Ag positive patients showed a median age of 8.8 ± 10.5 years old, while DENV-NS1-Ag negatives presented a median age of 20.7 ± 20.5 years old (*p* < 0.0001). This difference was observed in both males and females. RT-PCR was performed on 113 of 161 samples with 71% (80/113) of these samples being positive for active DENV infection (Table S1). We were able to identify the DENV serotype in 86% (69/80) of the samples, with DENV-2 being the most prevalent serotype (Table S2). Furthermore, fourteen samples presented a combination of two different serotypes: DENV-1/DENV-2 (11 samples), DENV2/DENV-4 (2 samples), and DENV 2/DENV 3 (1 sample).

### 3.2. Days of Symptoms Do Not Have a Significant Effect on RDT Results

To evaluate sensitivity and specificity of the DENV-NS1-Ag test in comparison to the RT-PCR, we only included 111 samples (2 samples were excluded for presenting symptoms after day 7). The DENV-NS1-Ag test presented a sensitivity of 85% (95%CI = 76% to 92%) and a sensitivity of 27% (95%CI = 15% to 44%) (Fisher exact test. *p* = 0.1116) when compared to the PCR as the gold standard (Table 2). 

The sensitivity of the NS1-Ag test was 87% (95%CI = 77% to 93%), and the specificity was 28% (95%CI = 15% to 46%) (Fisher exact test. *p* = 0.1282) if the DENV-NS1-Ag test was performed after 3 to 5 days of symptoms (Table 3), while the sensitivity decreased to 81% (95%CI = 57% to 93%) and specificity to 25% (95%CI = 13% to 70%) if the test was performed 6 to 7 days after presenting symptoms (Table 4).

### 3.3. NS1-Ag Positive Patients Present Lower Leukocyte Count Represented with a Significant Decrease in Monocytes 

Comparing the hematological findings between people with positive and negative DENV-NS1-Ag tests, we found no significant difference between groups with the exception of the leucocyte count (WBC), with a median leucocyte percentage of 3.26 × 10^3^/μL in the DENV-NS1-Ag positive group and 4.5 × 10^3^/μL in the DENV-NS1-Ag negative group (Mann–Whitney test, *p* = 0.0008) (Table 5). 

Additionally, leukopenia seems to be more severe in people with a positive NS1-Ag test (Chi-square test, *p* = 0.0043) (Table 6). 

**Figure 2:**
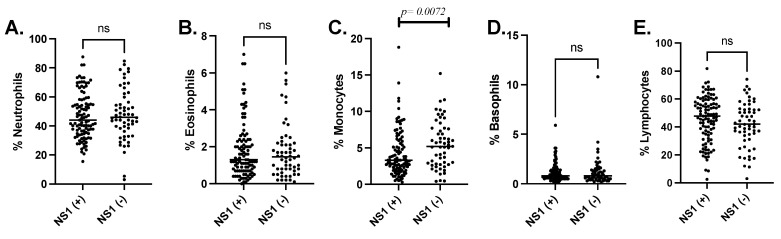
Leukocyte differential (**A**) Neutrophiles, (**B**) Eosinophils, (**C**) Monocytes, (**D**) Basophiles and (**E**) Lymphocytes reported in percentage of cells compared between patients with positive or negative DENV-NS1-Ag tests.

Comparison of the differential leukocyte proportions showed a significant decrease in monocytes in volunteers with a positive DENV-NS1-Ag test (Mann–Whitney test, *p* = 0.0072) (Figure 2). 

## 4. Discussion

Dengue is still a major arboviral disease of the tropics and subtropical areas and Latin America is no exception [27] Thus, it was is not surprising to find infections caused by each of the four DENV serotypes from the PCR analysis. Importantly, ~3% of infected individuals presented an infection with more than one serotype, consistent with previous reports showing Norte de Santander as a hyperendemic region for DENV transmission [30,31]. Although no other arboviruses were tested in our current study, our study area is well known for the transmission of other Aedes-borne viruses such as Zika virus (ZIKV) and Chikungunya virus (CHIKV). All of these viruses cause febrile disease, which are clinically indistinguishable during the first days of symptoms most of the time. Although no specific treatment is available against any of these diseases, accurate diagnosis is pivotal to prevent the progression of severity in all of them.

In Colombia, dengue fever is generally diagnosed using IgM-ELISA, however, not all health centers are properly equipped to offer this service to the public due to a lack of electricity or other necessary infrastructure, making it obligatory for physicians to learn how to differentiate these entities using clinical characteristics [32]. In this study we wanted to provide a list of hematological parameters found in people classified as a “probable” dengue infected patient using clinical and hematological parameters in wait of a confirmatory test. Our objective with this was to help guide decisions based on hematological parameters that are associated with a positive diagnostic test result. Our results suggest that, besides platelet count, total white blood cell count and monocyte cell count are important parameters differentiating the “probable dengue” cases with a positive confirmatory test from those with negative tests. In this context, other studies have described significant leukopenia in dengue patients [29,33], and although peripheral blood parameters change over the course of infection with DENV, in this study, we included volunteers seeking medical care 3 to 7 days after presenting symptoms compatible with DENV infection such as fever and rash. Once the physician sends the hematological tests, a patient is diagnosed as a “probable” dengue fever case based on the platelet and leucocyte counts as well as the hematocrit level. 

In the case of leukocyte count, we found that volunteers with a positive DENV-NS1-Ag test presented a significantly lower leukocyte count than those with probable dengue fever but with a negative RDT. Further analyzing the type of white blood cells that are more affected by DENV infection, we found that monocytes were significantly lower in people with a positive DENV-NS1-Ag test. However, monocytes are among the main targets of DENV infection and have been implicated in both protection and pathogenesis [34] because they produce inflammatory cytokines often involved with endothelial disruption and plasma leakage [35]. Previous studies suggest that monocytes are also the subject of apoptosis induced by DENV in several ways [36,37]. First, DENV infection in monocytes may induce pyroptosis, known as the induction of cell death, mediated by the concomitant activation of caspase 1 and interleukin 1 (IL1) production [37]. In addition, the production of tumor necrosis factor alpha (TNF-α) in DENV infected monocytes also induces apoptosis in such cells [36,37]. These mechanisms may explain the significant decrease in monocytes in people where the NS1 antigen can be detected by the RDT. Interestingly, a retrospective study in Taiwan with more than 1000 patients diagnosed with DENV 2 and 3 infections, reported lower absolute monocyte counts five days after fever onset in patients with mild disease when compared to patients with severe DENV infection [38]. Another study found that dengue fever patients presented significantly lower white blood cell and monocyte count when compared to patients with other febrile illness [39]. These studies highlight the importance of more research describing hematological findings in people infected with arboviruses in hyperendemic areas.

As explained above, a lack of testing for arbovirus infection in several primary health facilities results in these diseases often being diagnosed by clinical findings, including a decreased platelet count, leukopenia, and an increase in hematocrit among the main parameters [40,41]. Rapid diagnostic tests (RDT) can overcome some of the limitations posed by restricted access to electricity and highly trained personnel. Thus, we decided to test the efficacy of a DENV NS1-based RDT to identify DENV infection in these settings. Previous studies have determined that the presence of NS1 Ag overlays with the viremic phase of DENV infection during the first 4 to 5 days of clinical symptoms [42], but test performance may be affected by the time the person seeks medical attention. In this study, the NS1-Ag test presented a lower specificity than was previously reported in other studies; however, we found that the sensitivity of the DENV-NS1-Ag test in comparison to the RT-PCR decreased from 87% when the test was performed within the first 5 days of presenting symptoms to 81% if the test was performed afterwards. These results are consistent with previous studies showing the sensitivity and the specificity of the DENV-NS1-Ag test ranging between 27.8 and 93.4%, respectively [43,44,45]. 

Differences in the incidence of DENV infections between males and females have been previously reported. In this study, the majority of probable and confirmed dengue cases determined by the RDT were found among female volunteers. Additionally, a significant number of cases with a positive dengue diagnostic test were 15 years of age and younger, consistent with previous studies in the country [46]. Although previous studies have shown gender-associated differences in dengue fever incidence, the geographical area appears to strongly influence the associations and occurrence of dengue infections [47,48,49]. For instance, a study including dengue fever cases from at least six Asian countries showed a higher incidence of dengue in males [47], while other studies in Central and South America demonstrated a higher incidence of dengue in females [48,49].

A significant limitation in our current study is that we could not follow the patient’s clinical development or hematological findings on the days after the diagnosis so, it is impossible for us to correlate these parameters with progression to severe forms of infection. Another limitation is the lack of access to the previous dengue history of the patients or asymptomatic infections to compare hematological parameters, which would help to narrow down key factors associated with current infections.

In summary, our findings suggest a significant leukopenia in those with a positive DENV-NS1-Ag test and that monocytes may be the main white blood cells affected in this group of patients. Due to the presence of all four DENV serotypes and the probability of multiple infections in the area, it is important to evaluate diagnostic tools that can be implemented in areas where infrastructure does not allow the implementation of molecular tools such as RT-PCR. A vaccine against DENV in this area should be able to prevent infection against the four serotypes, especially in younger populations representing the majority of cases in this hyperendemic area of Colombia.

## Figures and Tables

**Table 1:** Epidemiological characteristics in patients with positive and negative DENV-NS1-Ag tests. Hospital Local de Los Patios, Norte de Santander, Colombia, 2018.

Variable	N	%	NS1-Ag Positive			NS1-Ag Negative			Mann-Whitney Test
			* n *	%	Median	* n *	%	Median	*p*-Value
Age (in years old)	161	100%	103	64%	8.8 ± 10.5	58	36%	20.7 ± 20.5	<0.0001 *
Female Age	92	57%	55	60%	9.9 ± 13.5	37	40%	22.4 ± 22.4	0.0016 *
Male Age	69	43%	48	70%	7.52 ± 5.0	21	30%	17.7 ± 17.0	0.0041 *
Children ≤ 15 years	126	78%	93	74%	6.2 ± 3.7	33	26%	6.9 ± 3.7	0.2972
Adults ≥ 15 years	35	22%	10	29%	32.2 ± 20.8	25	71%	38.8 ± 19.6	0.3158
Days of symptoms	161	100%	103	64%	4.9 ± 1.2	58	36%	4.7 ± 1.5	0.2458

* Statistical significant variables between patients with positive and negative DENV-NS1-Ag test.

**Table 2:** Comparison of samples tested by DENV-NS1-Ag test compared to PCR results (*n* = 111).

NS1-Ag	PCR		Total
	Positive	Negative	
Positive	67	24	91
Negative	11	9	20
Total	78	33	111

**Table 3:** Results of samples tested by DENV-NS1-Ag positive test compared to RT-PCR by days of symptoms.

≤5 Days	PCR			>6 Days	PCR		
NS1-Ag	Positive	Negative	Total	NS1-Ag	Positive	Negative	Total
Positive	54	21	75	Positive	13	3	18
Negative	8	8	16	Negative	3	1	4
Total	62	29	91	Total	18	4	22

**Table 4:** Dengue diagnostic tests results by days of symptoms.

Days of Symptoms	NS1-Ag (+)	NS1-Ag (−)	*n*	RT-PCR (+)	RT-PCR (−)	*n*
3	16	7	23	13	6	19
4	31	14	45	24	8	32
5	36	22	58	25	15	40
6	11	10	21	10	3	13
7	6	3	9	6	1	7
8	1	1	2	0	0	0
9	1	1	2	1	0	1
15	1	0	1	1	0	1
Total	103	58	161	80	33	113

**Table 5:** Hematological findings between people with positive and negative DENV-NS1-Ag tests. (*) indicate significant by Mann-Whitney test.

Parameter	NS1-Ag Positive (*n* = 103)	NS1-Ag Negative (*n* = 58)	Mann-Whitney Test
	Median (IQR)	Median (IQR)	*p* Value
Hemoglobin (g/dL)	12.9 (1.4)	13 (1.3)	0.6147
Hematocrit	39.0 (4.1)	39.9 (3.9)	0.3513
Platelet count (×10^9^/L)	113 (30.6)	108 (28.3)	0.4302
RBC (×10^12^/L)	4.5 (0.4)	4.59 (0.5)	0.2091
WBC (×10^9^/L)	3.26 (2.0)	4.52 (2.6)	0.0008 *
MCH (pg)	28.8 (2.7)	28.3 (2.7)	0.4452
MCV (ft)	85.8 (9.4)	84.8 (13.0)	0.4109
RDW CV%	12.8 (0.9)	12.8 (1.0)	0.1374
RDW-SD (fL)	46.4 (3.2)	45.8 (3.4)	0.7207

**Table 6:** Comparison of leukocytes count by DENV NS1-Ag test result.

Parameters	NS1-Ag Positive (*n* = 103)		NS1-Ag Negative (*n* = 58)	
Leukopenia (≤4.0 cells × 10^3^/μL)	67	65%	23	40%
Leukocytes (4.1–10 cells × 10^3^/μL)	35	34%	32	55%
Leukocytosis (>10 cells × 10^3^/μL)	1	1%	3	5%

## Data Availability

All data from this study is included in the current manuscript.

## Supplementary Materials

The following are available online at https://www.mdpi.com/article/10.3390/v13071401/s1. Table S1: PCR-based test results of samples with “probable dengue fever “diagnosis; Table S2: DENV serotype found in positive samples using RT-PCR.

## Abbreviations

Dengue virus (DENV); Enzyme-linked immune assay (ELISA); Non-structural protein 1 (NS1); Rapid diagnostic test (RDT); Polymerase chain reaction; Dengue hemorrhagic fever (DHF); Dengue shock syndrome (DSS); Pan-American Health Organization (PAHO); reverse transcription polymerase chain reaction (RT-PCR); Antigen (Ag); Ribonucleic acid (RNA).
