# Supplementary material for: Laboratory Findings in Patients with Probable Dengue Diagnosis from an Endemic Area in Colombia in 2018
Source: Viruses. 2021 Jul 19;13(7):1401. doi: 10.3390/v13071401 (PMC8310201; doi:10.3390/v13071401)
Supplement: Supplementary file 1 [file viruses-13-01401-s001.zip › viruses-1289970-supplementary.pdf]

Supplementary Tables

Table S1. PCR-based test results of samples with “probable dengue fever “diagnosis.

**RT-PCR**	**n**	**%**
Positive	80	71%
Negative	33	29%
**TOTAL**	**113**	100%

Table S2. DENV serotype found in samples positive by RT-PCR

**DENV serotype**	**n**	**%**
DENV 2	40	58%
DENV 1	24	35%
DENV 4	3	4%
DENV 3	2	3%
TOTAL	**69**	**100%**
